# An Institutional Perspective on the Number of Stent Retriever Pass and Rate of Recanalization in Mechanical Thrombectomy for Acute Ischemic Stroke: When to Stop?

**DOI:** 10.15388/Amed.2024.31.1.11

**Published:** 2024-02-27

**Authors:** Bheru Dan Charan, Shailesh B Gaikwad, Savyasachi Jain, Ajay Garg, Leve Joseph Devarajan Sebastian, M V Padma Srivastava, Rohit Bhatia, Awadh Kishore Pandit, Shashank Sharad Kale

**Affiliations:** 1 Department of Neuroimaging & Interventional Neuroradiology, All India Institute of Medical Sciences, New Delhi, India; 2 Department of Neurology, All India Institute of Medical Sciences, New Delhi, India; 3 Department of Neurosurgery, All India Institute of Medical Sciences, New Delhi, India

**Keywords:** Recanalization, ischemic stroke, mechanical thrombectomy, rekanalizacija, išeminis insultas, mechaninė trombektomija

## Abstract

**Background and purpose:**

Mechanical thrombectomy is the standard treatment modality for flow restoration in acute ischemic stroke. In cases of persistent occlusion, the optimal number of retrieval attempts before considering procedure termination is currently undetermined and is a topic for research. Therefore in this study, we studied the impact of the number of stent retrieval maneuvers on the recanalization of vessels.

**Methods:**

In this retrospective single-center observational study we included 52 patients with large vessel occlusion who underwent stent retriever mechanical thrombectomy. Successful recanalization rate was defined as modified TICI (Thrombolysis in Cerebral Infarction) 2b or 3.

**Result:**

The overall successful recanalization rate was 44.24%. The recanalization rate per stent retrieval attempt was the highest in 1st attempt (28.84%) and no recanalization was observed with the 3rd, 5th, and 6th attempts (p<0.001). At most 6 retrieval attempts were used.

**Conclusions:**

After two retrieval attempts, 91% of the patients were successfully recanalized and other after the 5th attempt could not result in recanalization.

## Abstract (it)

**Įvadas ir tikslas:**

Mechaninė trombektomija yra standartinis gydymo būdas, skirtas atkurti kraujotaką ūminio išeminio insulto atveju. Šiuo metu nėra nustatyta, koks yra optimalus trombų šalinimo bandymų skaičius prieš pradedant procedūrą, ir tai yra tyrimų objektas. Todėl šiame tyrime nagrinėjome stento ištraukimų skaičiaus įtaką kraujagyslių rekanalizacijai.

**Metodai:**

Šiame retrospektyviniame vieno centro stebėjimo tyrime dalyvavo 52 pacientai, kuriems buvo atlikta stento ištraukimo mechaninė trombektomija dėl didelių kraujagyslių okliuzijos. Sėkmingos rekanalizacijos rodiklis buvo apibrėžtas kaip modifikuoto TICI (Trombolizė esant smegenų infarktui) 2b arba 3.

**Rezultatas:**

Bendras sėkmingos rekanalizacijos rodiklis buvo 44,24 proc. Rekanalizacijos dažnis per vieną stento ištraukimo bandymą buvo didžiausias 1-ojo bandymo metu (28,84 %), o per 3-iąjį, 5-ąjį ir 6-ąjį bandymus rekanalizacijos nepastebėta (p<0,001). Buvo panaudoti ne daugiau kaip 6 ištraukimo bandymai.

**Išvados:**

Po dviejų ištraukimo bandymų 91 proc. pacientų buvo sėkmingai rekanalizuoti, o kitiems po 5-ojo bandymo rekanalizacijos pasiekti nepavyko.

## Introduction

Endovascular therapy (EVT) is widely acknowledged as the standard therapy for treating large-vessel occlusion in ischemic stroke. It is crucial to achieve successful reperfusion, as it significantly influences positive clinical outcomes [1]. Successful recanalization is defined as modified TICI (Thrombolysis in Cerebral Infarction) 2b or 3. Multiple attempts for clot retrieval are often necessary to achieve successful reperfusion. However, in cases of persistent occlusion, the optimal number of retrieval attempts before considering procedure termination is currently undetermined and is a topic for research.

There is still controversy in the literature regarding the number of retrieval attempts. Few studies describe the adverse effect of multiple retrievals on clinical outcomes [2,3], on the other hand, according to a recent study’s findings, the number of retrieval attempts necessary for achieving successful reperfusion does not serve as a predictor for positive clinical outcomes [4]. Hence, the impact of each successive retrieval on clinical outcomes remains uncertain.

In this retrospective study, our aim is to describe the efficacy of new-generation stent retriever devices to achieve reperfusion and the number of retrieval attempts in successful recanalization. Knowing of when to stop stent retrieval attempts in mechanical thrombectomy is necessary to prevent periprocedural complications.

## Material and method

Study design. We conducted a single-center retrospective study of prospectively collected data in our department from the data of Jan 2016 to Dec 2019. The number of stent retriever attempts in successful recanalization is a part of our study on predictors affecting successful recanalization.

We retrospectively scrutinized our RIS-PACS data of the patients who underwent mechanical thrombectomy from Jan 2016 to Dec 2020.

Patient selection criteria. Patients of 18–80 year old were included who underwent mechanical thrombectomy by stent retriever only. We included both in-hospital and emergency patients who suffered from occlusion of the ICA terminal, M1 or M2, or basilar artery. Patients who converted later into solumbra and aspiration technique were excluded from our study.

Data collection and analysis. We recorded patient age, number of attempts of stent retriever (solitaire, trevo) used and final recanalization grade according to the mTICI scale. The primary outcome of our study was successful angiographic recanalization (2b or 3). We categorized the patient on the basis of the number of stent retrieval attempts used in procedure. In each category defined by the number of attempts, we divided the patients into groups of successful and unsuccessful recanalization. Then the recanalization rate is compared with each category of number of attempts.

Statistical analysis. For statistical analysis with dichotomized groups of patients (consisting of patients with < or =2 and >2 attempts, or those with < or =3 and >3 attempts), an unpaired t-test was done to compare two group means and paired t-test for paired means within the group. The chi-square test was done to find the association between categorical variables, and the Fisher exact test was done if the expected cell count was less than 5. A p-value of less than 0.05 was considered significant.

## Result

A total of 52 patients were identified from our data system – those who underwent mechanical thrombectomy with stent retriever only. The mean age of the patients was 50.43 years; there were 22 males and 30 females. Successful recanalization was observed in 23 patients (46.12%) and unsuccessful recanalization in 29 (53.84%) patients. The maximum number of stent retriever attempts was 6, and it was used in 1 case only. In our study, the first stent retrieval attempt resulted in a successful recanalization in 15/52 (28.84%) patients (Table 1). In 1/52 patients, the intervention was aborted with persistent occlusion. In the remaining 36 patients, at least 1 further attempt was performed. As shown in Table 1, successful recanalization rates per stent retrieval attempt were highest for the first retrieval and lowest (0) for the third, fifth and sixth. Out of 23 patients who have successful recanalization, 15 patients were treated with 1 attempt, 6 patients with 2 attempts, and 2 patients were successfully recanalysed by the 4th attempt. After 2 stent retrieval attempts, successful recanalization was achieved in 21/23 patients (91.3 %) (p<0.001) and each subsequent attempt successfully produced only additional 8.7% of patients by 4th attempt, and more than 4 attempts did not result in recanalization. After all retrieval attempts, 23 (46.12%) patient were recanalized.

**Table 1:** Recanalization rate compared with number of attempts.

Final retrieval (No. of patients)	Total (52)		1st retrieval (n=52)		2nd retrieval (n=36)		3rd retrieval (n=24)		4th retrieval (n=10)		5th retrieval (n=3)		6th retrieval (n=1)	
TICI at end of procedure	2b/3	0/2a or aborted	2b/3	0/2a	2b/3	0/2a	2b/3	0-2a	2b/3	0-2a	2b/3	0-2a	2b/3	0-2a
Number of patients (n %)	23/52 (44.24)	29/52 (55.76)	15/52 (28.84)	1/52	6/36 (16.66)	6/36	0/24	14/24	2/10 (20%)	5/10	0/3	2/3	0/1	1/1

**Table 2:** Influence of the number of attempts of stent retriever on recanalization rate.

Treatment method	Attempts	Recanalization final				Fisher exact test P value
		Successful		Unsuccessful		
		N	%	N	%	
Stent only (52)	=/<2	21	91.3%	7	24.1 %	**<0.001**
	>2	2	8.7%	22	75.9%	
	Total	23	100%	29	100%	

We divided the attempts into two groups (=/<2 and >2). 21 patient (91.3 %) were recanalized by two attempts and the rest of 2 (8.7%) patients by more than two attempts. The number of attempts greater than 2 was associated with more unsuccessful recanalization, and this relation was statistically significant. (p <0.001).

## Discussion

In mechanical thrombectomy, there is still a lack of clarity regarding the optimal number of retrieval maneuvers to be used in situations where the clot cannot be readily extracted. In many cases, TICI 2b or 3 recanalization could not be achieved in single attempt. It is operator’s decisions, when to abort the thrombectomy procedure in technical difficulty where clot is not retrievable, and not based on empirical evidence. Therefor in this study we studied the impact of number of stent retrieval manoeuvres on recanalization of vessels.

Too many retrieval attempts lead to periprocedural complications such as SAH, ICH and vasospasms which deteriorates the outcome [5]. Too small number of attempts in technical difficulty leads to unsuccessful recanalization, so the optimal number of retrieval attempts should be studied. In our study, the fourth retrieval attempt showed potential for recanalization rate of 20% , which was comparable to a study which included MERCI retriever [6].

A large cohort study conducted by Fabian Flottmann reveals that successful reperfusion achieved by up to three retrieval attempts was significantly associated with good clinical outcome and the effect gradually decreased with increasing the number of attempts. [7] Examination of the recanalization rates with aggregated attempts corroborates our evidence that diminishing return appears to occur after 2 pulls.

The highest rate of successful recanalization was observed in 1st attempts (65%) while this rate was 26% by 2 attempts, as described in literature [2,7].

**Figure 1:**
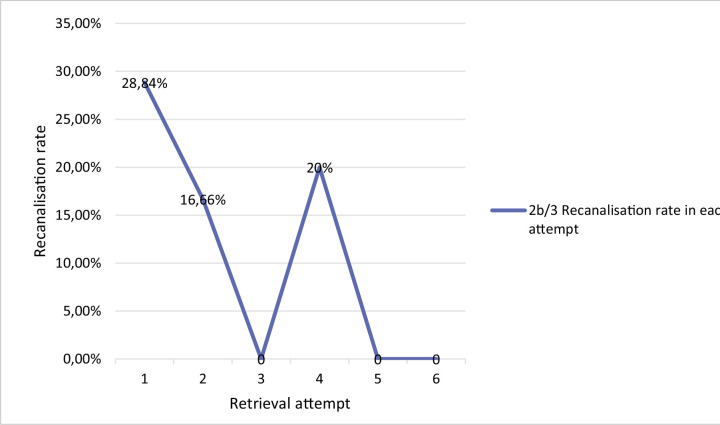
Recanalization rate per retrieval. Successful recanalization (2b/3) in all patients who underwent a certain number of retrieval attempts. TICI means Thrombolysis in Cerebral Infarction.

**Figure 2:**
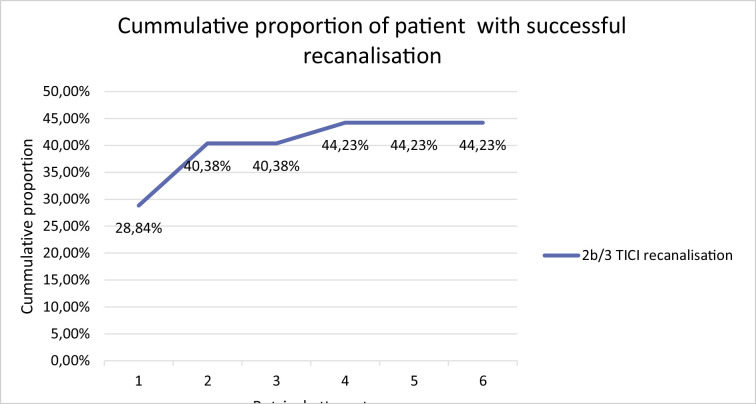
Cumulative proportion of patients with successful recanalization and of patients with good clinical outcome after each retrieval maneuver. TICI means Thrombolysis in Cerebral Infarction.

Additional research is required to ascertain the factors and underlying causes (such as time intervals and the potential risks of multiple retrieval attempts) contributing to sustained occlusions during instances of technical challenges.

## Limitations

Our study have several limitations, it is retrospective study with single center experience and small sample size. Clinical follow and modified ranking score was not available. Base line characteristics, pre MT imaging details were not included in this study. Many variables had missing data, which led to the exclusion of patients and selection bias. The reason of abandonment of procedure was under neurointerventionist control and due to retrospective nature, it could not be determined. We also did not elaborated the technical cause for failure of recanalization and termination of procedure, as this also was still unknown in previous literature [8]. Also the reason for multiple retrieval attempts, especially due to intracranial atherosclerosis, persistent occlusions, and reclusions, was not elaborated in our study and literature [9].

## Conclusions

Near ~ 91 % patients are recanalized during first two attempts and all further attempts (5 or more) did not result in recanalization. Further randomized controlled trials are needed to determine the optimal number of retrieval attempts.

## Availability of data and material (data transparency)

Yes
